# Timing of antibiotics, volume, and vasoactive infusions in children with sepsis admitted to intensive care

**DOI:** 10.1186/s13054-015-1010-x

**Published:** 2015-08-17

**Authors:** Bregje M. van Paridon, Cathy Sheppard, Garcia Guerra G, Ari R. Joffe, for the Alberta Sepsis Network

**Affiliations:** Department of Pediatrics, Sophia Childrens Hospital Erasmus University Medical Centre, Rotterdam, The Netherlands; Faculty of Nursing, University of Alberta, Edmonton, AB Canada; Department of Pediatrics, Division of Pediatric Critical Care Medicine, University of Alberta, Edmonton, AB Canada; 4-546 Edmonton Clinic Health Academy, 11405 87 Ave, Edmonton, AB T6G 1C9 Canada

## Abstract

**Introduction:**

Early administration of antibiotics for sepsis, and of fluid boluses and vasoactive agents for septic shock, is recommended. Evidence for this in children is limited.

**Methods:**

The Alberta Sepsis Network prospectively enrolled eligible children admitted to the Pediatric Intensive Care Unit (PICU) with sepsis from 04/2012-10/2014. Demographics, severity of illness, and outcomes variables were prospectively entered into the ASN database after deferred consent. Timing of interventions were determined by retrospective chart review using a study manual and case-report-form. We aimed to determine the association of intervention timing and outcome in children with sepsis. Univariate (*t*-test and Fisher’s Exact) and multiple linear regression statistics evaluated predictors of outcomes of PICU length of stay (LOS) and ventilation days.

**Results:**

Seventy-nine children, age median 60 (IQR 22–133) months, 40 (51 %) female, 39 (49 %) with severe underlying co-morbidity, 44 (56 %) with septic shock, and median PRISM-III 10.5 [IQR 6.0-17.0] were enrolled. Most patients presented in an ED: 36 (46 %) at an outlying hospital ED, and 21 (27 %) at the Children’s Hospital ED. Most infections were pneumonia with/without empyema (42, 53 %), meningitis (11, 14 %), or bacteremia (10, 13 %). The time from presentation to acceptable antibiotic administration was a median of 115.0 [IQR 59.0-323.0] minutes; 20 (25 %) of patients received their antibiotics in the first hour from presentation. Independent predictors of PICU LOS were PRISM-III, and severe underlying co-morbidity, but not time to antibiotics. In the septic shock subgroup, the volume of fluid boluses given in the first 2 hours was independently associated with longer PICU LOS (effect size 0.22 days; 95 % CI 0.5, 0.38; per ml/kg). Independent predictors of ventilator days were PRISM-III score and severe underlying co-morbidity. In the septic shock subgroup, volume of fluid boluses in the first 2 hours was independently associated with more ventilator days (effect size 0.09 days; 95 % CI 0.02, 0.15; per ml/kg).

**Conclusion:**

Higher volume of early fluid boluses in children with sepsis and septic shock was independently associated with longer PICU LOS and ventilator days. More study on the benefits and harms of fluid bolus therapy in children are needed.

**Electronic supplementary material:**

The online version of this article (doi:10.1186/s13054-015-1010-x) contains supplementary material, which is available to authorized users.

## Introduction

Severe sepsis in children is increasing, associated with significant mortality, and can be followed by significant neurocognitive sequelae [1–5]. The surviving sepsis guidelines aim to improve outcomes in children with severe sepsis, and recommend resuscitation for signs of shock with fluid boluses followed by vasoactive infusions, and appropriate antibiotics, each delivered within the first hour of presentation [6, 7]. Although a ubiquitous intervention, the evidence for dosing of fluid bolus therapy (FBT) in septic shock is of very low quality [8]. The evidence for FBT in children is predominantly based on two observational studies finding improved outcomes with aggressive FBT in children with septic shock [9, 10]. Other observational studies in children have concluded that bundles for recognition and early resuscitation of septic shock that include aggressive FBT may reduce mortality and hospital length of stay (LOS) [11–16]. However, the role of FBT resuscitation is unclear in most of these studies [11, 12, 14, 15], and others have limitations including having unusually high mortality [15, 16], an unclear definition of “early fluid resuscitation” [16], and retrospective patient identification [12–14, 16].

There is also evidence that each hour of delayed antibiotic therapy in adults with septic shock is associated with increasing mortality [17, 18]. Although this is theoretically compelling, there is only limited evidence for this early antibiotic administration in children with sepsis [19, 20]. Finally, the evidence for timing of vasoactive infusions is even more limited; in adults, there are data to suggest that starting at 1–6 h after onset of septic shock, or by 14 h after onset of septic shock, is associated with lower mortality [21, 22].

The Alberta Sepsis Network (ASN) prospectively enrolled a cohort of children with sepsis admitted to the only two pediatric intensive care units (PICUs) in Alberta, Canada. In this study we retrospectively reviewed the charts of children enrolled at the Stollery Children’s Hospital PICU to determine timing of antibiotic administration, and in the septic shock subgroup, timing of fluid boluses and vasoactive infusions. The objective of this study was to determine whether there is an association between timing of these interventions and outcomes in children with sepsis. We hypothesized that early antibiotics, volume, and vasoactive infusions would be associated with fewer days of ventilation and shorter LOS in the PICU.

## Methods

### Ethics

This study was approved by the Health Research Ethics Board of the University of Alberta (Pro00008797), and all enrolled patients gave deferred signed informed consent for participation. Deferred consent allowed enrolment as early as possible in PICU, followed by informed consent to use blood work and continue in the study within 3 days of inclusion.

### The Alberta sepsis network

The ASN prospectively enrolled all eligible children up to age 17 y who were admitted to the two PICUs in Alberta with a diagnosis of sepsis, between April 2010 and March 2014. Sepsis was defined as systemic inflammatory response syndrome (SIRS) caused by a suspected or proven bacterial or fungal infection, with antibiotics prescribed, and an arterial and/or central venous line in place [23]. The requirement for an arterial and/or central venous line was to facilitate study blood work and justify deferred consent. Patients were excluded if they were not expected to survive ≥24 h, were refusing intubation or vasoactive infusions (i.e., palliative care), or if they had already had severe sepsis for ≥48 h (defined as sepsis with cardiovascular dysfunction, acute respiratory distress syndrome, or two other organ dysfunctions). Demographic, infection, and severity of illness variables (including pediatric logistic organ dysfunction (PELOD), and pediatric risk of mortality (PRISM)-III scores) were recorded prospectively [24, 25]. Site of infection was defined as that diagnosed by the attending medical team. Septic shock was defined as having an infusion of an inotrope or vasopressor (dopamine, dobutamine, epinephrine, norepinephrine, or milrinone) started (i.e., a new vasoactive agents started, or a dose change of a vasoactive agent) on the first calendar day of sepsis. This was a pragmatic definition, meant to identify children who almost certainly required FBT according to current guidelines. Severe underlying co-morbidity was defined as having a cardiac, neurological, or at least two other organ systems involved in a chronic disease prior to onset of sepsis.

### Retrospective data collection

The Stollery Children’s Hospital has the only PICU serving Northern Alberta, much of Northern British Columbia, Yukon, the North West Territories and Nunavut, and is the largest referral center for cardiac surgery, extracorporeal life support, and solid organ transplantation for Western Canada. The charts of all enrolled patients in our PICU were reviewed to determine the following information: 1) time of presentation with sepsis: the admission time to the emergency department (ED), or if onset was on the hospital ward or PICU, the time when there was both new fever with a temperature >38.2 °C and a blood culture had been sent. Both fever and blood culture were required in the hospitalized PICU or ward patients because those patients can have recurrent fever, and performing a blood culture was thought to signify that a new episode of sepsis was suspected; and 2) antibiotic administration time: the antibiotic(s) administered fulfill the predefined criteria for the type of infection. This time is at the start of the infusion of intravenous antibiotic (or time when given enterally, for ciprofloxacin, metronidazole, or trimethoprim-sulfamethoxazole). If more than one antibiotic is required by the predefined criteria, the time is the start of the second antibiotic. In patients with septic shock, the following was also recorded: 1) fluid bolus time: time from presentation with sepsis to the first fluid bolus of at least 20 ml/kg (the usually suggested individual FBT volume) of isotonic intravenous fluid, including crystalloids (normal saline, Ringer’s lactate, plasmalyte), and/or colloids (5 % albumin, plasma); 2) volume of fluid boluses in first 2 h after presentation (to reflect a pragmatic definition of early FBT in resuscitating septic shock); 3) volume of fluid boluses given from presentation until first vasoactive infusion was started; and 4) vasoactive infusion time: time from presentation with sepsis to start of the first intravenous vasoactive infusion.

A case report form and study manual were created for data collection, definitions, and a predefined list of acceptable antibiotic(s) for each site of infection (Additional file 1). This list was based on the published Bugs & Drugs handbook [26], and expanded to include antibiotic choices that were not the local first choice, but that could be expected to be acceptable (i.e., antibiotics that are broader spectrum than required for that infection) [20]. The expanded list was prepared by one of the authors (ARJ) who is a pediatric infectious diseases specialist.

### Statistics

Descriptive results are presented as proportions (percentages), mean (standard deviation), or median (IQR). The primary outcome was the association between timing of antibiotic administration and PICU LOS. In the septic shock subgroup, the main outcomes of interest were the association between timing of antibiotic administration and the amount of early fluid boluses, and PICU LOS. Predefined potential predictors of outcome were: 1) demographic variables: age (in months), and severe underlying co-morbidity; 2) severity of illness measures: admission day PRISM-III and PELOD scores; 3) timing variables in the entire cohort: time to appropriate antibiotic(s); appropriate antibiotic(s) given within 1 h; and 4) timing variables in the septic shock subgroup: time to vasoactive infusion; vasoactive infusion within 3 h; time to volume bolus of 20 ml/kg; volume of boluses given in first 2 h; volume boluses of ≤20 ml/kg in first 2 h; and volume of boluses given prior to the start of the first vasoactive infusion. Predefined outcomes were: 1) ventilator days; 2) PICU LOS in days; 3) delta-PELOD: the drop in PELOD from day 1 to 3 of PICU admission, as an indicator of improvement in organ dysfunction(s); and 4) mortality by 1 y after the index sepsis admission. Univariate comparisons were performed using the *t* test for independent samples and Fisher’s exact test. Multiple linear regression analysis was used to determine adjusted effect sizes for predictor variables of continuous outcomes. Multiple logistic regression was used to determine association between potential predictors with mortality by 1 y after the index sepsis admission. Age, PRISM-III score, and severe underlying co-morbidity were entered into all regression analyses, and we planned to enter time to antibiotic(s), volume boluses given in the first 2 h, and volume boluses given prior to vasoactive infusion into the regressions, unless univariate analysis suggested other timing variables were statistically significant and should replace these variables. For all analyses, a two-sided *p* value ≤0.05 was considered significant.

## Results

### Description of the cohort

**Fig. 1:**
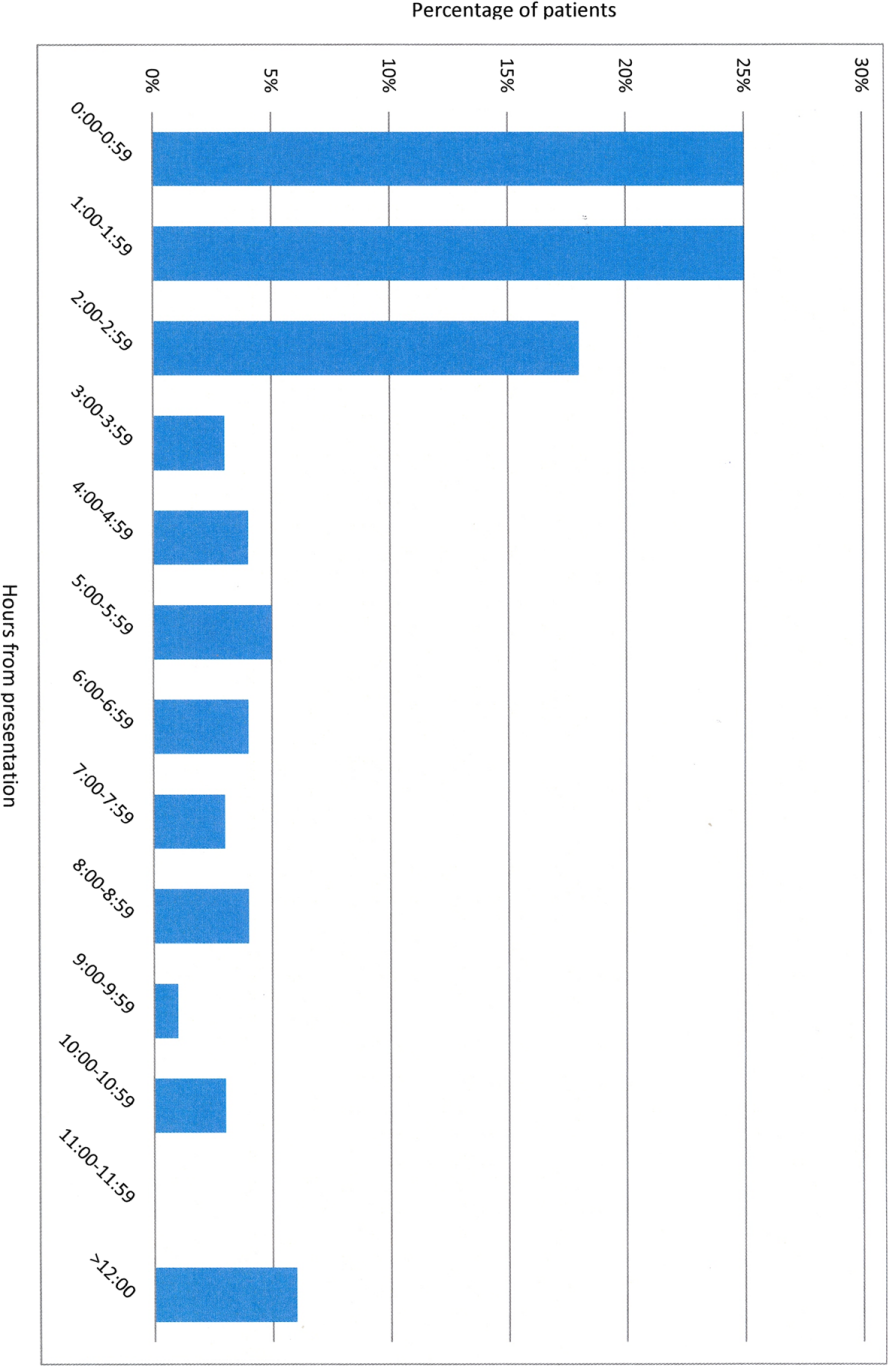
Time to administration of appropriate antibiotics after presentation with sepsis

Of 83 patients enrolled in the ASN database, 4 were excluded due to lost charts, with no way to determine timing of interventions. Of 79 children included, the median age was 60 (IQR 22–133) months, 40 (51 %) were female, 39 (49 %) had severe underlying co-morbidity, and the median PRISM III score on the day of sepsis was 10.5 (6.0–17.0). Mortalities by 1 y after the sepsis admission numbered 5/79 (6.3 %); only one of these occurred during the hospitalization, the others at 5, 6, 11, and 12 months after the admission. Most patients presented in an ED: 36 (46 %) at an outlying hospital ED, and 21 (27 %) at the our Children’s Hospital ED. Only 2 (3 %) had developed sepsis while on our Children’s Hospital wards, and 20 (25 %) while in the PICU. Most infections were pneumonia with/without empyema (n = 42, 53 %), meningitis (n = 11, 14 %), or bacteremia (n = 10, 13 %); other sites included intra-abdominal (n = 4, 5 %), urinary tract (n = 3, 4 %), cellulitis (n = 2, 3 %), and other (n = 7, 9 %). Median time from presentation to acceptable antibiotic administration was 115.0 (IQR 59.0–323.0) minutes; 20 patients (25 %) received their antibiotics during the first hour after presentation (Fig. 1). There were 44 patients (56 %) with septic shock, of whom 3 (6.8 %) died by 1 y after the sepsis admission.

### Univariate analyses

The association between antibiotic administration within 1 h of presentation and demographics and outcomes is shown in Table 1. Early administration of antibiotics was associated with higher PRISM-III score, and longer PICU LOS. Among the patients with septic shock, the association between volume ≤20 ml/kg given in the first 2 h and demographics and outcomes is shown in Table 1. Administration of higher-volume boluses was associated with older age and higher PRISM-III score, with no difference in outcomes.

**Table 1:** Univariate associations with early (within 1 h) appropriate antibiotic therapy and with early therapy with bolus volume over 20 ml/kg in children with sepsis and septic shock, respectively

Variable	Abx 0–1 h	Abx >1 h	*P* value	≤20 ml/kg in 2 h	>20 ml/kg in 2 h	*P* value
Number	20	59		26	18	
Age	69 (60)	79 (66)	0.56	56 (52)	120 (62)	0.001
PRISM	15 (7)	11 (7)	0.03	11 (7)	17 (8)	0.006
PELOD	15 (8)	17 (10)	0.44	16 (9)	21 (12)	0.090
Severe underlying disease	11/20 (55 %)	28/59 (47 %)	0.61	15/26 (58 %)	9/18 (50 %)	0.76
ΔPELOD	3.4 (8.4)	6.0 (11.6)	0.35	3.2 (12.7)	10.0 (9.9)	0.067
Ventilator days	10.4 (9.2)	7.1 (8.9)	0.17	6.5 (4.8)	9.1 (7.8)	0.18
PICU days	19.5 (21.2)	10.2 (10.1)	0.01	10.7 (7.4)	17.2 (22.4)	0.17

Comparisons were performed using the *t* test for independent samples, and Fisher’s exact test as appropriate. Values are presented as mean (standard deviation) or proportion (percent). *Abx* appropriate antibiotic therapy, *PRISM* pediatric risk of mortality score, *PELOD* pediatric logistic organ dysfunction score, *PICU* pediatric ICU

The association between the potential predictors and dichotomous outcomes of ventilator days >7, PICU LOS >7 days, and delta-PELOD > median are shown in Table 2. FBT in those ventilated for at least 7 days was mean 36.7 (SD 34.9 ml/kg) compared to FBT in those ventilated for <7 days of mean 20.2 (SD 17.6), *p* = 0.05 (Table 2). The association between potential predictors and mortality by 1 y after admission are shown in Table 3. The FBT volume given in the first 2 h was similar according to outcomes of number of PICU days, delta PELOD from day 1 to 3, and mortality by 1 y (Tables 2 and 3).

**Table 2:** Univariate analysis for predictors of prolonged ventilation and PICU length of stay, and for drop in PELOD score between days 1 to 3 of sepsis

Variable	Ventilated 1–7 d	Ventilated >7 d	*P* value	PICU 0–7 d	PICU >7 d	*P* value	Δ PELOD ≤ median	Δ PELOD > median	*P* value
Number	45	19		33	46		40	35	
Age, months	82 (62)	62 (66)	0.17	90 (64)	67 (63)	0.12	60 (65)	94 (59)	0.022
PRISM	10.4 (6.1)	14.5 (8.6)	0.02	10.3 (5.5)	13.0 (8.3)	0.11	11.5 (6.6)	12.7 (7.5)	0.45
PELOD	14.4 (8)	20.7 (11)	0.007	14.4 (8.6)	17.8 (10.4)	0.13	-	-	
Severe underlying disease	18/45 (40 %)	19/29 (66 %)	0.056	11/33 (33 %)	28/46 (61 %)	0.022	22/40 (55 %)	14/35 (40 %)	0.25
Delta PELOD	6.9 (8.4)	2.7 (14.0)	0.12	8.1 (8.4)	3.4 (11.9)	0.06	-	-	
Time to Abx	243 (306)	235 (359)	0.92	223 (296)	243 (336)	0.79	234 (329)	239 (325)	0.95
Abx in first hour	10/45 (22 %)	9/29 (31 %)	0.43	5/33 (15 %)	15/46 (33 %)	0.12	13/40 (33 %)	7/35 (20 %)	0.30
Time to inotropes	546 (395)	428 (342)	0.32	654 (368)	428 (359)	0.056	533 (421)	493 (344)	0.74
Inotropes within 3 h	6/24 (25 %)	5/18 (28 %)	0.66	2/15 (13 %)	9/29 (31 %)	0.27	6/20 (30 %)	4/22 (18 %)	0.34
Time to 20 ml/kg volume	225 (247)	132 (179)	0.22	212 (220)	185 (252)	0.75	233 (306)	152 (167)	0.32
Volume in 2 h	20.2 (17.6)	36.7 (34.9)	0.05	23 (18.9)	29 (30.2)	0.48	23 (23)	33 (30)	0.27
Volume ≤20 ml/kg in 2 h	16/24 (67 %)	9/18 (50 %)	0.35	10/15 (67 %)	16/29 (55 %)	0.53	14/20 (70 %)	10/22 (45 %)	0.098
Volume to inotropes	35.2 (32.1)	46.7 (34.4)	0.27	41 (35)	39 (32)	0.88	37 (28)	46 (36)	0.35
Ventilator days	-	-		-	-		10.3 (11.2)	5.8 (5.1)	0.036
PICU days	-	-		-	-		15.8 (17.8)	9.7 (8.1)	0.071

Comparisons were performed using the *t* test for independent samples, and Fisher’s exact test as appropriate. Values are given as mean (standard deviation) or proportion (percent); times are given in minutes. *PICU* pediatric ICU, *PELOD* pediatric logistic organ dysfunction score, *Abx* antibiotic therapy

**Table 3:** Univariate associations with mortality by one year after the index sepsis admission

Variable	Alive (n = 74)	Dead (n = 5)	*P* value
Time to appropriate antibiotics, minutes	247 (325)	45 (30)	0.17
Time to inotrope, minutes	495 (350)	652 (711)	0.74
Volume in 2 h, ml/kg	29 (27)	7 (12)	0.18
Volume to inotrope, ml/kg	42 (32)	17 (29)	0.20
Age, months	80 (65)	25 (26)	0.004
PRISM	11.8 (7.4)	12.0 (5.9)	0.96
PELOD day 1	16.5 (9.7)	15.2 (12.0)	0.78
Severe underlying co-morbidity	35/74 (47 %)	4/5 (80 %)	0.20

Data were analyzed using the *t* test for independent samples and Fisher’s exact test as appropriate. Values are given as mean (standard deviation) or proportion (percent). *PRISM* pediatric risk of mortality score; *PELOD* pediatric logistic organ dysfunction score

### Multiple regression analyses

Using multiple logistic regression analysis the independent predictors of ventilator days, PICU LOS, and delta-PELOD from day 1 to 3 are shown in Table 4, for both the overall cohort, and for the septic shock subgroup. Independent predictors of PICU LOS were PRISM-III score, and severe underlying co-morbidity, but not time to antibiotics. In the septic shock subgroup, the volume of fluid boluses given in the first 2 h was independently associated with longer PICU LOS (effect size 0.22 days; 95 % CI 0.5, 0.38, per ml/kg; *p* = 0.01). Independent predictors of ventilator days were PRISM-III score and severe underlying co-morbidity. In the septic shock subgroup, volume of fluid boluses in the first 2 h was also independently associated with more ventilator days (effect size 0.09 days; 95 % CI 0.02, 0.15, per ml/kg; *p* = 0.009). The only independent predictor of the delta-PELOD was age (effect size 0.042; 95 % CI 0.004, 0.080; *p* = 0.032), and in the septic shock subgroup there were no independent predictors. Using multiple logistic regression, there were no independent predictors for mortality by 1 y after admission in the entire cohort, or the septic shock subgroup.

**Table 4:** Multiple linear regression analysis of independent predictors of outcomes in children with sepsis, and with septic shock

Variable	PICU days			Ventilator days		
	Effect size	95 % CI	*P* value	Effect size	95 % CI	*P* value
PRISM	0.64	0.23, 1.04	0.003	0.30	0.01, 0.59	0.04
Age	0.015	-	0.89	−0.02	-	0.22
Severe underlying disease	7.27	1.34, 13.2	0.017	4.1	-	0.05
Time to antibiotics, minutes	−0.010	-	0.92	0.002	-	0.45
Model R2	15.2 %			7.4 %		
Subgroup: septic shock						
PRISM	0.71	0.16, 1.25	0.012	0.24	0.03, 0.45	0.024
Age	0.05	-	0.30	−0.22	-	0.12
Severe underlying disease	0.29	-	0.06	3.82	0.57, 1.07	0.023
Time to antibiotics, minutes	-.067	-	0.63	0.049	-	0.72
Volume in 2 h	0.22	0.05, 0.38	0.010	0.09	0.02, 0.15	0.009
Volume prior to inotropes	−0.19	-	0.38	−0.13	-	0.54
Model R2	23.4 %			32.1 %		

Analyses were performed using stepwise multiple linear regression, except for ventilator days in the entire cohort, where the variables were forced into the model. For the drop in pediatric logistic organ dysfunction score (PELOD) from day 1 to 3 of sepsis, the only independent predictor in the entire cohort was age (effect size 0.042; 95 % CI 0.004, 0.080; *p* = 0.032); for the septic shock subgroup, there were no independent predictors. *PRISM* pediatric risk of mortality score

## Discussion

We retrospectively reviewed the timing of antibiotics, fluid, and vasoactive agents in children who were prospectively enrolled in the ASN database from the PICU for Northern Alberta. The main findings are the following. First, early timing of appropriate antibiotics was associated with longer PICU LOS on univariate analysis; however, it was not independently associated with PICU LOS, ventilator days, or change in PELOD score from day 1 to 3 in all included children (n = 79) or those with septic shock (n = 44). This is despite fairly wide variability in time to administration of appropriate antibiotics in the patients (median 115.0; IQR 59.0 − 323.0 minutes). Second, higher volume of fluid boluses in the first 2 h of presentation with sepsis was independently associated with longer PICU LOS and more ventilator days in children with septic shock. Of note, time to starting vasoactive agents was not associated with outcomes. Third, severity of illness (PRISM-III score) and severe underlying co-morbidity were independently associated with longer PICU LOS and more ventilator days. Fourth we did not identify independent predictors of mortality by 1 y after the index sepsis admission. These results are contrary to our initial hypotheses, which were that early appropriate antibiotics, and more volume bolus resuscitation, would be associated with improved outcomes.

The evidence for aggressive FBT has been questioned; two systematic reviews found that FBT may be harmful in children [27, 28]. These results are largely driven by the randomized controlled FEAST trial performed in the developing world in which FBT led to increased mortality from cardiovascular collapse, regardless of presentation syndrome or initial response to fluid therapy [29–32]. Two single-center retrospective observational studies suggest improved outcome in 9 and 24 children receiving more FBT for septic shock; however, these studies involved only 34 and 91 children with septic shock who had survived to PICU admission, were inotrope-dependent, and had a pulmonary artery catheter in situ [9, 10]. In the larger study, children who had signs of shock that resolved quickly were included in the appropriate fluid therapy group; the median fluid volumes given to children who died (32.9 ml/kg) were higher than those given to survivors (20 ml/kg) [10, 30]. Theoretically, FBT could cause harm by several mechanisms: rapid reduction in sympathetically mediated compensatory mechanisms; treatment-induced hyperchloremic metabolic acidosis; ischemia-reperfusion injury; fluid overload; and endothelial glycocalyx degradation [8, 31–33]. The endothelial glycocalyx has important functions in regulating vasomotor tone, oncotic gradient, endothelial porosity, microvascular thrombosis, oxidative stress, and endothelial adhesion of platelets, red blood cells, and white blood cells [34]. It is surprising that no evidence exists for the effect of fluid boluses on outcomes more than a few hours after the bolus [8]. Contrary to the initial study by Rivers et al. [35], three large well-conducted randomized controlled trials of early goal-directed therapy (EGDT) in septic shock in adults suggest that the EGDT group received more fluid, inotrope, and blood transfusion than the standard care group, yet had either equivalent or worse outcomes [36–39]. Our finding that FBT was independently associated with longer PICU LOS and ventilator days, without improving the change in PELOD score between days 1 and 3 of septic shock, is compatible with these findings.

Alternatives to FBT may include slower infusion of volume, or earlier use of vasoactive infusions. The evidence for timing of vasoactive agents in children is weak. In adults, recent observational studies have found an improved survival if vasoactive agents were started in hours 1–6, or by hour 14 after onset of septic shock [21, 22]. The effect may be confounded by the level of blood pressure that is aimed for, and the evidence for how low a level of blood pressure is too low is being questioned [40]. Nevertheless, in our study, we found no association between timing of vasoactive agents and outcomes in children with septic shock. There are limitations to this finding, however. First, we did not determine the time of onset of hypotension, and that is likely a better indicator of when to start vasoactive agents than time from presentation with sepsis. Second, we had a small cohort of children with septic shock (n = 44). Third, because of the small cohort, we did not analyze outcomes according to individual agents; it is possible that one agent may be better than another in determining outcome, although this has not been shown in adult studies [41, 42].

Early administration of antibiotics has been associated with better outcome in septic shock and ICU patients with infection in many adult observational studies [17, 43–45]. Each hour of delayed antibiotic administration from onset of hypotension has been associated with increased mortality, although a recent study suggested this may only start after 4 h [17, 22]. This suggests a paradigm of septic shock where the bacterial load must be reduced early and quickly in order to prevent the sequelae of uncontrolled infection [18]. Recent data in children suggest early antibiotics are independently associated with improved outcomes in septic shock, particularly after a cutoff of 3 h [18, 19]. We did not find this effect in our cohort. Nevertheless, we do not suggest that antibiotics can be safely delayed in children with sepsis, due to limitations of our study.

This study has limitations. This is a single-center cohort of children admitted to PICU with sepsis and an arterial and/or central venous line who consented to enrollment in the ASN database in which extra blood work was performed for research purposes; thus, not all children with sepsis were included. It is possible that the inclusion criteria may have missed children who responded quickly to FBT, and thus, did not require PICU admission or an arterial/central line. The number of children included is modest (n = 79), particularly in the septic shock subgroup (n = 44). The septic shock subgroup included only children who went on vasoactive infusions, and may have missed children who had aggressive FBT alone. The onset of hypotension was not recorded, and therefore, we cannot determine timing of interventions from that event. Not all patients had microbiologically confirmed infection, and thus, it is possible that some of the patients may have had non-infection causes of severe systemic inflammatory response syndrome. The mortality in hospital (1/79, 1.3 %) and 1 y after the sepsis hospitalization (5/79, 6.3 %) was low, suggesting this may have been a less sick cohort of PICU sepsis patients. Finally, the timing of interventions was recorded retrospectively from chart review. Nevertheless, this is a cohort of PICU patients with clinically diagnosed sepsis and reflects the real-world situation in managing these patients. We pre-specified primary and secondary outcomes, and the analysis plan. In addition, the PRISM and PELOD scores of enrolled patients were comparable to published pediatric trials with higher mortality (Additional file 2: Table E1), suggesting the cohort comprised patients with high severity of illness.

## Conclusions

Administration of a higher volume of early fluid boluses in children with sepsis and septic shock was independently associated with longer PICU LOS and ventilator days. There was no adverse effect on outcomes with administration of early appropriate antibiotics and early vasoactive infusions. This small single-center observational study cannot prove cause and effect, and is hypothesis-generating only. Our results are not sufficient to suggest a change in clinical practice. Nevertheless, we believe that the results suggest that more study on the benefits and harms of FBT in children with sepsis are needed to better inform management.

## Key messages

- Early timing of appropriate antibiotics was not independently associated with PICU LOS, ventilator days, or change in PELOD score from days 1 to 3 in all included children (n = 79) or those with septic shock (n = 44)
- Higher volume of fluid boluses in the first 2 h of presentation with sepsis was independently associated with longer PICU LOS and more ventilator days in children with septic shock
- More study on the benefits and harms of fluid bolus therapy in children is needed, as there are multiple theoretical reasons why fluid boluses may be harmful

## Additional files

Additional file 1:
**The case report form and study manual for the chart review.** This file provides the definitions used, and the acceptable antibiotics for the different types of infection. (PDF 2280 kb) Additional file 2: Table E1. Comparison of patient severity to recent studies of children in intensive care. This file describes the severity of illness in the Alberta Sepsis Network (ASN) cohort and its comparability to several recent studies of critically ill children. (PDF 1529 kb)

## Abbreviations

ASN: Alberta Sepsis Network
ED: emergency department
EGDT: early goal-directed therapy
FBT: fluid bolus therapy
IQR: interquartile range
LOS: length of stay
PELOD: pediatric logistic organ dysfunction score
PICU: pediatric intensive care unit
PRISM: pediatric risk of mortality score
SIRS: systemic inflammatory response syndrome
