# Supplementary material for: Timing of antibiotics, volume, and vasoactive infusions in children with sepsis admitted to intensive care
Source: Crit Care. 2015 Aug 17;19(1):293. doi: 10.1186/s13054-015-1010-x (PMC4539944; doi:10.1186/s13054-015-1010-x)
Supplement: Additional file 1: — The case report form and study manual for the chart review. This file provides the definitions used, and the acceptable antibiotics for the different types of infection. (PDF 2280 kb) [file 13054_2015_1010_MOESM1_ESM.pdf]

Timing of antibiotics, volume, and vasoactive infusions in children with sepsis admitted to intensive care.

ESM_1_Joffe:

The case report form and study manual used for the chart review.

Authors: Bregje M van Paridon BSc[1], Cathy Sheppard RN[2], Gonzalo Garcia Guerra MSc MD[3], Ari R Joffe MD[3], for the Alberta Sepsis Network.

Affiliations: 1. Erasmus University Medical Center, Rotterdam, the Netherlands; 2. Faculty of Nursing, University of Alberta, Edmonton, Alberta, Canada; 3. Department of Pediatrics, Division of Pediatric Critical Care Medicine, University of Alberta, Edmonton, Alberta, Canada

Corresponding author: Ari R Joffe MD; 4-546 Edmonton Clinic Health Academy; 11405 87 Ave; Edmonton, Alberta, Canada, T6G 1C9. Email: ari.joffe@albertahealthservices.ca Phone: 780 2485435. Fax: 888 7901283

CRF: The Timing of Intervention for Severe Sepsis in Children

ASB study number: STO________________

1. Age in months: ______________

2. Location of presentation: ☐ER ☐An outside Hospital ☐Stollery Ward ☐Stollery PICU

3. Site of infection:
   - ☐Meningitis
   - ☐Bacteremia: ☐GPC Clumps CONS
     - ☐GPC Clumps S aureus
     - ☐GPC chains
     - ☐GNB
     - ☐Candida
     - ☐Other
   - ☐Pneumonia ☐Community
     - ☐Nosocomial
   - ☐Empyema
   - ☐Abdominal
   - ☐UTI
   - ☐Fascitis
   - ☐Mediastinits
   - ☐Other

4. Times:
   - 4A. Presentation: ______________________
   - 4B. Acceptable Antibiotic(s) Time: ____________________
   - 4C. Interval to acceptable antibiotic: ___________ (hr:min)

For Septic Shock Subgroup: those put on inotropes on Day 1 of ASN inclusion:

5. Times for fluid:
   - 5A. First fluid bolus time: ________________
   - 5B. Interval to first bolus time: ___________ (hr:min)
   - 5C. Fluid used: ☐NS ☐RL ☐Plasmalyte ☐FFP ☐PRBC ☐5% albumin
   - 5D. Total volume given in first 2 hours: __________ ml/kg

6. Times for inotropes:
   - 6A. First inotrope start time: _______________
   - 6B. What was first inotrope: ☐dopamine ☐dobutamine ☐epinephrine ☐norepinephrine ☐milrinone ☐phenylephrine ☐vasopressin
   - 6C. Interval to first inotrope time: ___________(hr:min)
   - 6D. Total volume given before first inotrope start time: ____________ ml/kg

ASN SEPSIS AND TIME OF ANTIBIOTICS AND FLUID BOLUS STUDY: GLOSSARY

Site of infection	Acceptable empiric antibiotic
Meningitis (community acquired)	Cefotaxime,<br>Ceftriaxone,<br>Meropenem,<br>Ceftazidime<br><1month: also accept- Ampicillin and Gentamicin. If proven Listeria [growth from CSF], then only need Ampicillin alone.
Pneumonia	
-community	> 3mos: Ampicillin<br>Any age: any of below<br>Cefuroxime<br>Cefotaxime<br>Ceftriaxone<br>Piperacillin-tazobactam<br>Meropenem<br>Imepenem<br>Levofloxacin<br>If proven MRSA [from blood culture, endotracheal aspirate, or empyema fluid]:<br>Vancomycin<br>Linezolid
-nosocomial	Piperacillin-tazobactam<br>Meropenem<br>Imepenem<br>Ciprofloxacin<br>If proven MRSA [from blood culture, endotracheal aspirate, or empyema fluid]:<br>Vancomycin<br>LInezolid
Empyema	Same as for community acquired pneumonia
Bacteremia	
-GPC clumps: CONS	Vancomycin
-GPC clumps: S.aureus	Vancomycin<br>Cloxacillin<br>Piperacillin-tazobactam<br>Meropenem<br>Imepenem<br>Cefotaxime

	Ceftriaxone<br>Clindamycin<br>If proven MRSA:<br>Vancomycin<br>Linezolid
-GPC chains:<br>Streptococcus sp.<br>Enterococcus sp.	Vancomycin<br>Piperacillin-tazobactam<br>Piperacillin<br>Meropenem<br>Impenem<br>Ampicillin<br>Penicillin
-GNB<br>E coli, Klebsiella sp,<br>Pseudomonas sp,<br>Enterobacter sp,<br>others	Piperacillin-tazobactam<br>Gentamicin<br>Tobramycin<br>Meropenem<br>Imepenem<br>Ciprofloxacin<br>Aztreonam
-Candida sp.	Amphotericin: B, lipid complex, Abelcet, Ambisome<br>Echinocandin: caspofungin, micafungin<br>Fluconazole
Abdominal: appendicitis, bowel perforation or surgery, abscess, trauma	Ampicillin and Gentamicin/Tobramycin<br>Clindamycin and Gentamicin/Tobramycin<br>Ciprofloxacin and Metronidazole<br>Piperacillin-tazobactam<br>Meropenem<br>Imepenem<br>Ertapenem<br>Ceftriaxone/Cefotaxime and Metronidazole
UTI	Ampicillin (community acquired)<br>Gentamicin<br>Tobramycin<br>Piperacillin<br>Piperacillin-tazobactam<br>Meropenem<br>Imepenem<br>TMP/SMX (if sensitive)<br>Ceftriaxone<br>Cefotaxime<br>If proven Enterococci [on urine culture]:<br>Not to count: gentamicin, tobramycin, TMP/SMX, Ceftriaxone, or Cefotaxime
Fascitis	Penicillin and Cefotaxime/Gentamicin/Tobramycin

	Clindamycin and Cefotaxime/Gentamicin/Tobramycin<br>Piperacillin-tazobactam<br>Meropenem<br>Imepenem<br>If proven Group A Streptococcus [Group A Streptococcus, or Streptococcus pyogenes on blood culture or surgical specimen from fascitis]:<br>Penicillin<br>Clindamycin<br>If proven MRSA [from same specimens as above]:<br>Vancomycin<br>Linezolid
Mediastinitis	One of Vancomycin/Cloxacillin/Ancef<br>AND/OR:<br>Piperacillin/tazobactam<br>Gentamicin<br>Tobramycin<br>Imepenem<br>Meropenem<br>Ciprofloxacin

Miscellaneous less common sites:

a. Mastoiditis	same as for meningitis<br>Piperacillin/tazobactam<br>Imipenem
b. Cellulitis	same as for fascitis
c. Bacterial Tracheitis	same as for pneumonia (except for ampicillin)
d. Burns	same as for mediastinitis (i.e. nosocomial flora)
e. Retropharyngeal abscess	same as for pneumonia
f. Unknown source (community acquired)	same as for meningitis

Definitions:

Fluid bolus:
-NS, RL, Plasmalyte, 5% albumin, PRBC, FFP, Platelets
-20ml/kg

Time of presentation:
-ER: admission time to ER.
-ward or PICU: time that the following have occurred- new fever >38.2 and blood culture sent.

Antibiotic time:
-fulfills the criteria: intravenously (can be enterally for: ciprofloxacin, metronidazole, TMP/SMX), appropriate choice for site
-time: at the start of the infusion; if two antibiotics are required, then at the start time of the infusion for the second antibiotic.

Septic shock:
-inotropes (dopamine, dobutamine, epinephrine, norepinephrine, phenylephrine, or milrinone) started on day 1 of ASN.

Sepsis:
-as in ASN: SIRS due to confirmed or suspected infection

Nosocomial infection:
-the infection develops more than 72 hours after hospital admission

Community acquired infection:
-the infection is present on admission to hospital, or incubating on admission to hospital [defined as the infection developing within 72 hours of hospital admission].
