# Supplementary material for: Timing of antibiotics, volume, and vasoactive infusions in children with sepsis admitted to intensive care
Source: Crit Care. 2015 Aug 17;19(1):293. doi: 10.1186/s13054-015-1010-x (PMC4539944; doi:10.1186/s13054-015-1010-x)
Supplement: Additional file 2: Table E1. — Comparison of patient severity to recent studies of children in intensive care. This file describes the severity of illness in the Alberta Sepsis Network (ASN) cohort and its comparability to several recent studies of critically ill children. (PDF 1529 kb) [file 13054_2015_1010_MOESM2_ESM.pdf]

Timing of antibiotics, volume, and vasoactive infusions in children with sepsis admitted to intensive care.

ESM_2_Joffe:

A comparison of patient severity in this Alberta Sepsis Network cohort to severity in recent studies of children in intensive care.

Authors: Bregje M van Paridon BSc[1], Cathy Sheppard RN[2], Gonzalo Garcia Guerra MSc MD[3], Ari R Joffe MD[3], for the Alberta Sepsis Network.

Affiliations: 1. Erasmus University Medical Center, Rotterdam, the Netherlands; 2. Faculty of Nursing, University of Alberta, Edmonton, Alberta, Canada; 3. Department of Pediatrics, Division of Pediatric Critical Care Medicine, University of Alberta, Edmonton, Alberta, Canada

Corresponding author: Ari R Joffe MD; 4-546 Edmonton Clinic Health Academy; 11405 87 Ave; Edmonton, Alberta, Canada, T6G 1C9. Email: ari.joffe@albertahealthservices.ca Phone: 780 2485435. Fax: 888 7901283

Table E1. Comparison of patient severity to recent studies of children in intensive care.

Trial	PRISM	PELOD	Mortality
TRIPICU (restrictive group)[1]	9.6 (6.7)	6.3 (6.8)	14/320 (4.4%)
VIP (vasopressin group)[2]	14 [10-20]	11.7 [7.3-20.5]	10/35 (30%)
Adrenal (AI group)[3]	6 [3-10]	11 [1-12]	4/115 (3.5%)
Han et al.[4]	13 [6-24]	-	26/91 (29%)
Current study	10.5 [6.0-17.0]	16.4 (9.8);<br>12 [11, 22].	1/79 (1.3%)

AI: adrenal insufficiency; PELOD: pediatric logistic organ dysfunction score (as mean (standard deviation) or median [interquartile range]); PRISM: pediatric risk of mortality III score (as mean (standard deviation) or median [interquartile range]); TRIPICU: Transfusion in Pediatric Intensive Care Unit randomized trial; VIP: Vasopressin in Pediatric Vasodilatory Shock randomized trial.

References:

1. Lacroix J, Hebert PC, Hutchison JS, Hume HA, Tucci M, Ducruet T, Gauvin F, Collet J-P, Toledano BJ, Robillard P, Joffe A, Biarent D, Meert K, Peters JM, for the TRIPICU Investigators, the Canadian Critical Trials Group, and the Pediatric Acute Lung Injury and Sepsis Investigators Network: **Transfusion strategies for patients in pediatric intensive care units.** *NEJM*, 2007;**356:**1609-1619.
2. Choong K, Bohn D, Fraser DD, Gaboury I, Hutchison JS, Joffe AR, Litalien C, Menon K, McNamara P, Ward RE, for the Canadian Critical Care Trials Group: **Vasopressin in pediatric vasodilatory shock: a multicenter randomized controlled trial.** *Am J Respir Crit Care Med*, 2009;**180(7):**632-639.
3. Menon K, Ward RE, Lawson ML, Gaboury I, Hutchison JS, Hebert PC: **A prospective multicenter study of adrenal function in critically ill children.** *Am J Respir Crit Care Med,* 2010;**182(2):**246-251.
4. Han YY, Carcillo JA, Dragotta MA, Bills DM, Watson RS, Westerman ME, Orr RA: **Early reversal of pediatric-neonatal septic shock by community physicians is associated with improved outcome.** *Pediatrics,* 2003;**112(4):**793-799.
